# Body Composition in Elite Soccer Players from Youth to Senior Squad

**DOI:** 10.3390/ijerph18094982

**Published:** 2021-05-07

**Authors:** Marijan Spehnjak, Marko Gušić, Slavko Molnar, Mario Baić, Slobodan Andrašić, Musa Selimi, Draženka Mačak, Dejan M. Madić, Suzana Žilič Fišer, Goran Sporiš, Nebojša Trajković

**Affiliations:** 1 Archdiocese of Zagreb, 10000 Zagreb, Croatia; m.spehnjak@yahoo.com (M.S.); macak.md@yahoo.com (D.M.); dekimadic@gmail.com (D.M.M.); 2 Faculty of Sport and Physical Education, University of Novi Sad, 21000 Novi Sad, Serbia; gusicmarko@yahoo.com (M.G.); molslavko@gmail.com (S.M.); 3 Faculty of Kinesiology, University of Zagreb, 10000 Zagreb, Croatia; mario.baic@kif.unizg.hr (M.B.); goran.sporis@kif.unizg.hr (G.S.); 4 Faculty of Economics, University of Novi Sad, 24000 Subotica, Serbia; andrasicslobodan@yahoo.com; 5 Faculty of Physical Education and Sport, University of Prishtina, 10000 Prishtina, Kosovo; musa.selimi@uni-pr.edu; 6 FERI, University of Maribor, 2000 Maribor, Slovenia; suzana.zilicfiser@um.si; 7 Faculty of Sport and Physical Education, University of Nis, 18000 Nis, Serbia

**Keywords:** soccer, age, body size, body composition, reference intervals

## Abstract

There is a strong relationship between body composition and performance in male soccer players. This study aimed to display an optimal body height and weight, and body composition profile of male soccer players for four competitive age groups. This cross-sectional study included four groups: U-15 (*n* = 152), U-17 (*n* = 154), U-19 (*n* = 61), and seniors (*n* = 27). Body height and weight were measured under standard conditions, and the bioelectrical impedance analyzer (BIA) analyzed body composition. On average, soccer players in the U-15 group had significantly lower body height, weight, body mass index, skeletal muscle mass, fat-free mass, total body water and basal metabolic rate than U-17, U-19 and seniors, but a higher percentage of body fat than U-17 and U-19, *p* < 0.05. In addition, the results show significant non-linear increases in body height, weight and body composition as the age of soccer players increases, with the exception of the percentage of body fat mass, which tends to significantly decrease with age. The main findings of this study are that body fat mass remains stable and similar across all age groups, including in the senior squad.

## 1. Introduction

Success in sports has been associated with specific morphological characteristics [1]. This was confirmed by Gardasevic et al. [2]. Although Gardasevic et al. also stated that morphological characteristics and body composition are one of the most important characteristics for complex sports games such as soccer [2], soccer players change their morphological content during the season. Therefore, monitoring should be conducted in order to design training cycles in the right order [3]. Moreover, research concerning the impact of morphological characteristics on soccer performance is not straightforward, mainly because of the playing positions, but also because of the individual characteristics of players [4]. Nevertheless, monitoring of body composition can help players to improve their performance and evaluate applied training plan results [5], which is an important part of the training process.

According to Reilly et al., there is a strong relation between the body composition and fitness of male soccer players [6]. However, caution is advisable when making direct comparisons between level, age, position and sex, as elite males tend to have a higher ratio of fat-free mass to body fat mass, which may adversely affect the endurance of players. Even though Ingebrigtsen et al. found no significant effect of playing position on anthropometric measures [7], Krustrup et al. found that defenders tend to be taller and heavier [8], and that midfielders tend to have the lowest body fat percentage compared to the other playing positions. Moreover, Leão et al. highlighted the increases in height and weight and decreases in the percentage of body fat mass with age, within the expected ranges [9]. Milsom et al. stated that percentage of body fat mass was lower in the first team (10.0 ± 1.6) compared with both U-21 (11.6 ± 2.5, *p* = 0.02) and U-18 (11.4 ± 2.6, *p* = 0.01) players [10]. However, they stated that the difference was not due to variations in fat mass between squads, but rather the presence of more fat-free mass in the first team and U-21 compared with U-18 players. 

Although age and body composition are strongly associated, there is no clear consensus as to whether this relationship is positive or negative. Leão et al. found increases in fat-free mass and decreases in fat mass with advancing age [9]; however, Manna, Khanna and Dhara found the opposite in similar populations [11].

It has been reported that soccer coaches select young players based on their anthropometric characteristics and body composition rather than their technical and tactical performance [12]. Moreover, the majority of studies have focused largely on players of 11–16 years of age with possibly the greatest impact of biological maturation [13]. In contrast, there are few data for older adolescent players, which is the last competitive age group before players face the challenges associated with the highest competitive levels in the sport.

Morphological characteristics are used in training monitorization as an important determinant of performance. However, there are differences in the literature regarding estimation procedures. Moreover, although soccer players differ from the general population regarding their body composition, very little difference is expected between professional players at clubs. Specifically, due to changes in training methodology in recent years, there are possible differences in age groups in soccer. The similarities in training methodology should imply that players will maintain an appropriate body composition while progressing from younger to senior groups. We wanted to find out if similarities in training methodology in recent years have contributed to some important changes in the body composition profile of soccer players. Moreover, once soccer players compete in elite senior professional competition, factors other than anthropometry and body composition determine whether they attain elite status. So, it was important to see if some changes occur between younger selection and senior squads. Therefore, the aim of this study was to describe the morphological characteristics of a large group of soccer players, across different age groups. 

## 2. Materials and Methods 

In this cross-sectional study, the sample of male soccer players included 394 subjects who were allocated into 4 groups according to age: U-15 (*n* = 152), U-17 (*n* = 154), U-19 (*n* = 61) and seniors (*n* = 27). All soccer players in this sample were affiliated to a professional soccer club and completed, on average, 10 h per week of combined soccer training and competition. All participants had a similar diet (Club canteen), as well as a controlled hydration level 24 h before testing. The experimental protocol was approved by the ethical committee of the institutional ethics committee from the Faculty of Sport and Physical Education, University of Novi Sad. The study was conducted in accordance with the Declaration of Helsinki and following the ethical standards of the University of Novi Sad (number: 42/2017).

Body height was measured under standard conditions. The InBody 230 body composition analyzer (InBody Co. Ltd., Cerritos, CA, USA) was utilized to measure body weight and body fat percentage (BF%), by applying the bioelectrical impedance method. The InBody 230 is a segmental impedance device, with a tetrapolar eight-point tactile electrode method using 20 and 100 kHz frequencies for each body segment. This method presents a sum of complex procedures which exclude the possibility of making errors or inaccuracies. InBody 230 is an accurate and convenient bioelectrical impedance analysis (BIA) instrument for measuring weight, total body water, lean body mass, lean mass (dry), muscle mass (skeletal), body fat mass, BMI, percentage of body fat, basal metabolic rate (BMR), segmental analysis of lean body mass (right and left arm, trunk, right and left leg) and impedance of each body segment. After the InBody 230 was turned on and warmed up, the instrument processed the self-calibration method with self-testing in zero weight conditions, followed by an adjustment of the internal circuit. After calibration, the loadcell was set to zero kilograms. Any pressure or weight could cause inaccurate calibration during the self-calibration process. The analysis and data output was carried out according to the manufacturer’s algorithm, including the BF% equation (Percentage of Body Fat (%) = Body Fat Mass/Body Weight × 100) and BMI (Weight in kilograms)/(Height in meters)^2^. The InBody 230 impedance was measured while the participants were in a standing position and with hands holding grips. InBody 230 is a reliable device for soccer players in the current study with high ICC for percentage of body fat (≥0.98) and low SEM. The standardized protocol was applied according to the InBody 230 User Manual [14]. The subjects entered the testing area and removed their shoes and socks and wore only light clothing. Once they were positioned on the InBody 230, their age, sex, and stature were entered. The InBody 230 displays a visual cue (photo) indicating how and when to hold the handles during the impedance measure. We registered the following body composition variables: body mass index, skeletal muscle mass, percentage of body fat mass, fat-free mass, total body water and basal metabolic rate.

Response variables (morphological characteristics) and age are presented as a mean and standard deviation (SD) for each group of soccer players. Each group of soccer players covers two years (U-15, U-17, U-19), except for the senior group (>18 years old). A one-way analysis of variance was used to check whether groups of soccer players differed in body composition and anthropometric measures. Tukey’s post hoc test was used for the group pairwise comparisons.

The relationships between the groups of soccer players, and anthropometric and BIA measures were estimated using Spearman’s rank correlation coefficient. Additionally, we investigated whether the body composition and anthropometric measures depended on the soccer players’ group using second- or third-order polynomial regression. Separate investigation into body composition and anthropometric measures in the function of the soccer players’ group involved testing several curve-fitting and smoothing techniques for the mean and standard deviation estimation. A cubic regression model estimated the mean body height in the function of the soccer players’ group, while the means of weight, body mass index, skeletal muscle mass, body fat mass (%), fat-free mass, total body water and basal metabolic rate were estimated by the fitted values from the quadratic regression models. The goodness of fit for each polynomial regression model was carefully assessed by the coefficient of determination (R^2^ × 100) and standard error of estimate (SEE) are reported. The standard deviations were estimated using the polynomial regression functions of the absolute residuals. Afterwards, the coefficients from those models were multiplied by a corrective constant equal to √(*π*/2) = 1.253.

We used Altman’s method of absolute scaled residuals to estimate the centiles for anthropometric and body composition measures and to develop the growth curves [15]. Assuming that the measures have a Gaussian distribution with a mean and a standard deviation and that, in general, both vary smoothly with the groups of soccer players, the centile curves (5th, 10th, 90th, and 95th) were calculated using Altman’s formula:

$$Centile=mean\left(soccer\;age\;\mathrm{rank}\right)\;\pm \;k\times SD\left(soccer\;age\;\mathrm{rank}\right)$$

where *k* is the corresponding centile of Gaussian distribution, mean is the mean, and *SD* is the standard deviation of the mean of the body composition and anthropometric measurements for each group of soccer players. The level of significance was set at *p* ≤ 0.05. Statistical analysis was conducted in SPSS (v.20, IBM Corporation, Armonk, NY, USA). The growth diagrams were created in Microsoft Excel 2016.

## 3. Results

### 3.1. The Anthropometric and Body Composition Measures across the U-15, U-17, U-19 and Seniors

Soccer players in the U-15, U-17, U-19 and senior groups significantly differed, on average, in all anthropometric and body composition measures, except in kilograms of body fat mass (*p* = 0.19). The U-15 group had significantly lower values for almost all anthropometric and body composition measures as compared to the remaining groups. Although the U-15 group had a significantly higher percentage of body fat mass than the U-17 and U-19 groups, the U-15 group did not significantly differ from the seniors in the percentage of body fat mass. However, the U-17, U-19 and seniors did not differ in any of the observed measures. For detailed results of a one-way analysis of variance, see Table 1.

### 3.2. Regression Models of Anthropometric and Body Composition for the U-15, U-17, U-19 and Seniors

We found that all study outcomes (body height, body weight, BMI, skeletal muscle mass, body fat mass, fat-free mass, total body water, basal metabolic rate) tend to significantly increase with the increased age groups of soccer players, except for the percentage of body fat mass, which tends to decrease. Correlation coefficients between each outcome and age category are presented in Table 2.

Body height (F_(2, 393)_ = 43.43, SEE = 10.49), weight (F_(2, 393)_ = 98.40, SEE = 10.20), body mass index (F_(2, 393)_ = 51.20, SEE = 2.17), skeletal muscle mass (F_(2, 393)_ = 110.83, SEE = 5.76), percentage of body fat mass (F_(2, 393)_ = 13.13, SEE = 5.14), fat-free mass (F_(2, 393)_ = 108.40, SEE = 9.65), total body water (F_(2, 393)_ = 90.51, SEE = 7.31) and basal metabolic rate (F_(2, 393)_ = 106.24, SEE = 206.60) significantly depended on the soccer players’ group. However, body fat mass did not significantly depend on the age group in this sample of soccer players (F = 3.57, *p* = 0.06, R^2^ = 0.01, SEE = 3.41). Table 3 presents regression equations for the mean and SD of anthropometric and body composition measures in the function of soccer age rank (U-15, U-17, U-19 and seniors) in males and its corresponding coefficient of determination.

**Figure 1:**
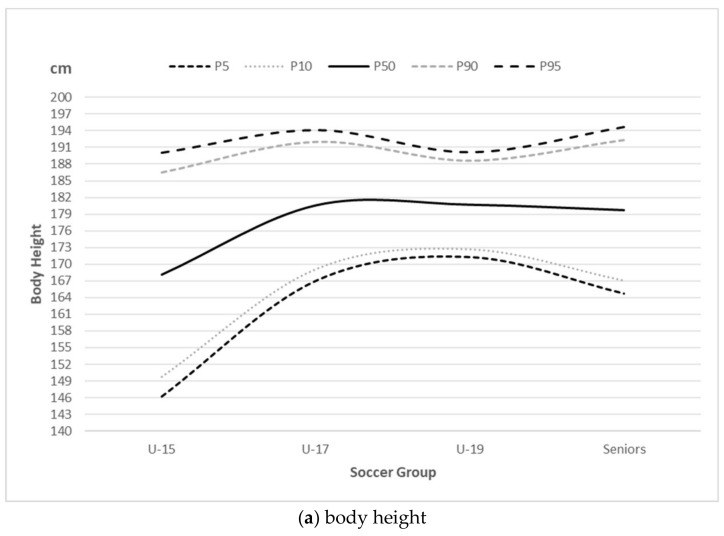

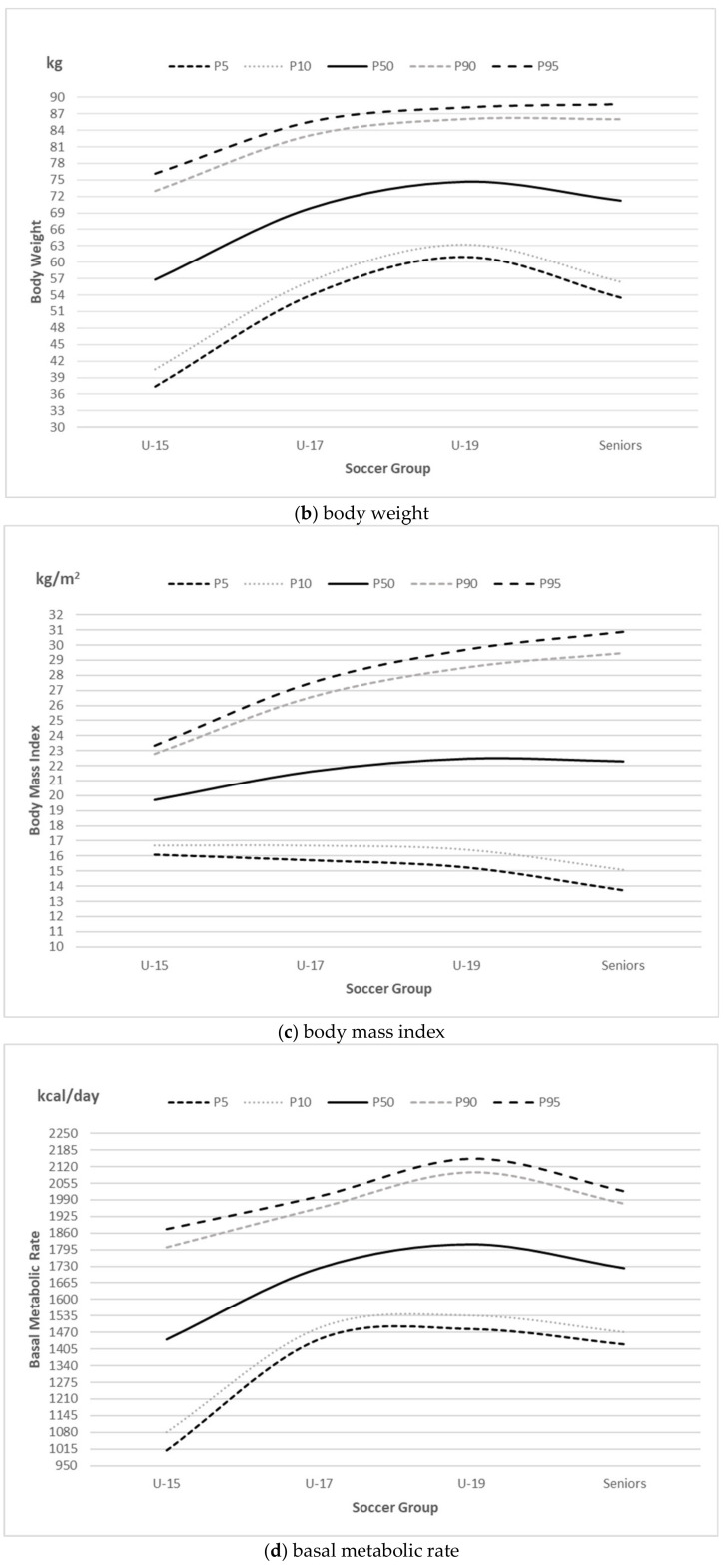

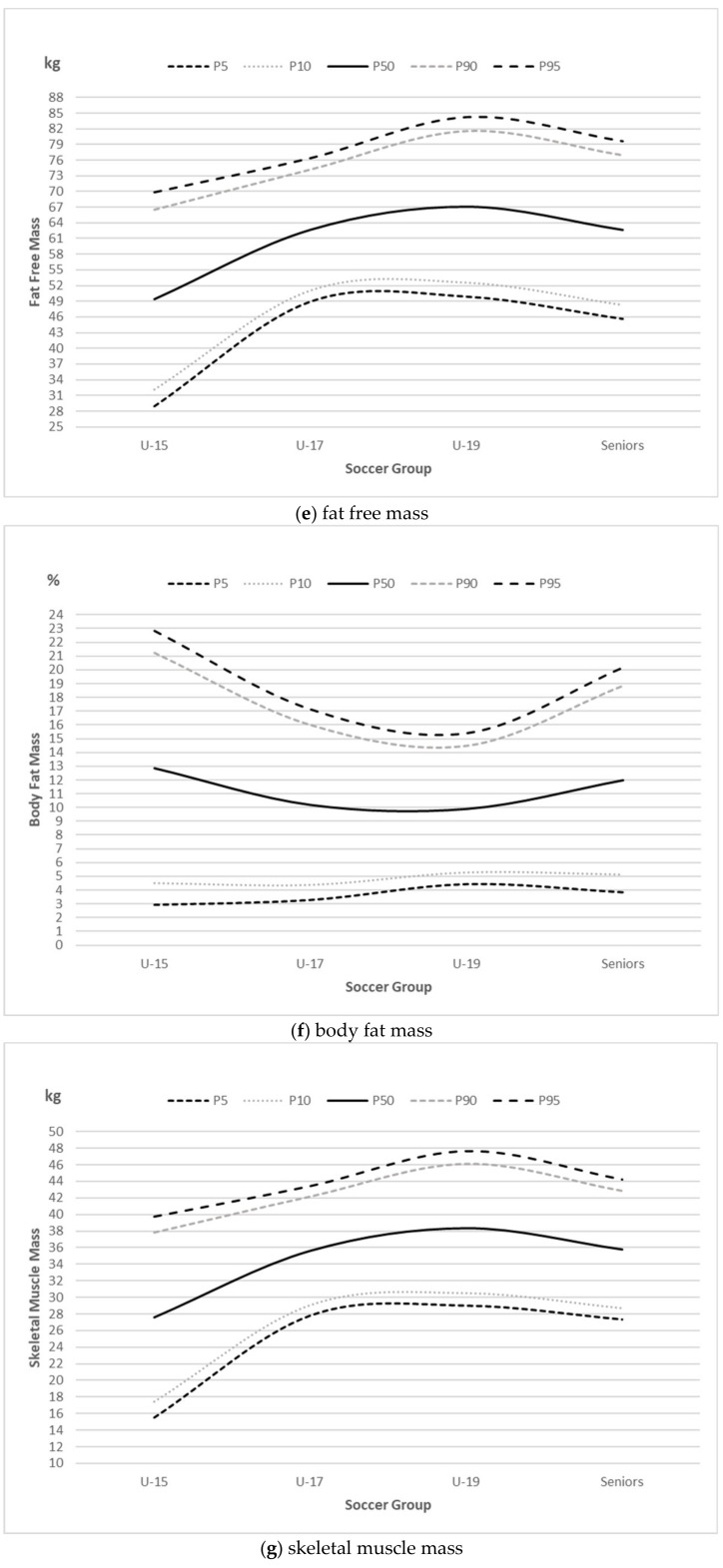

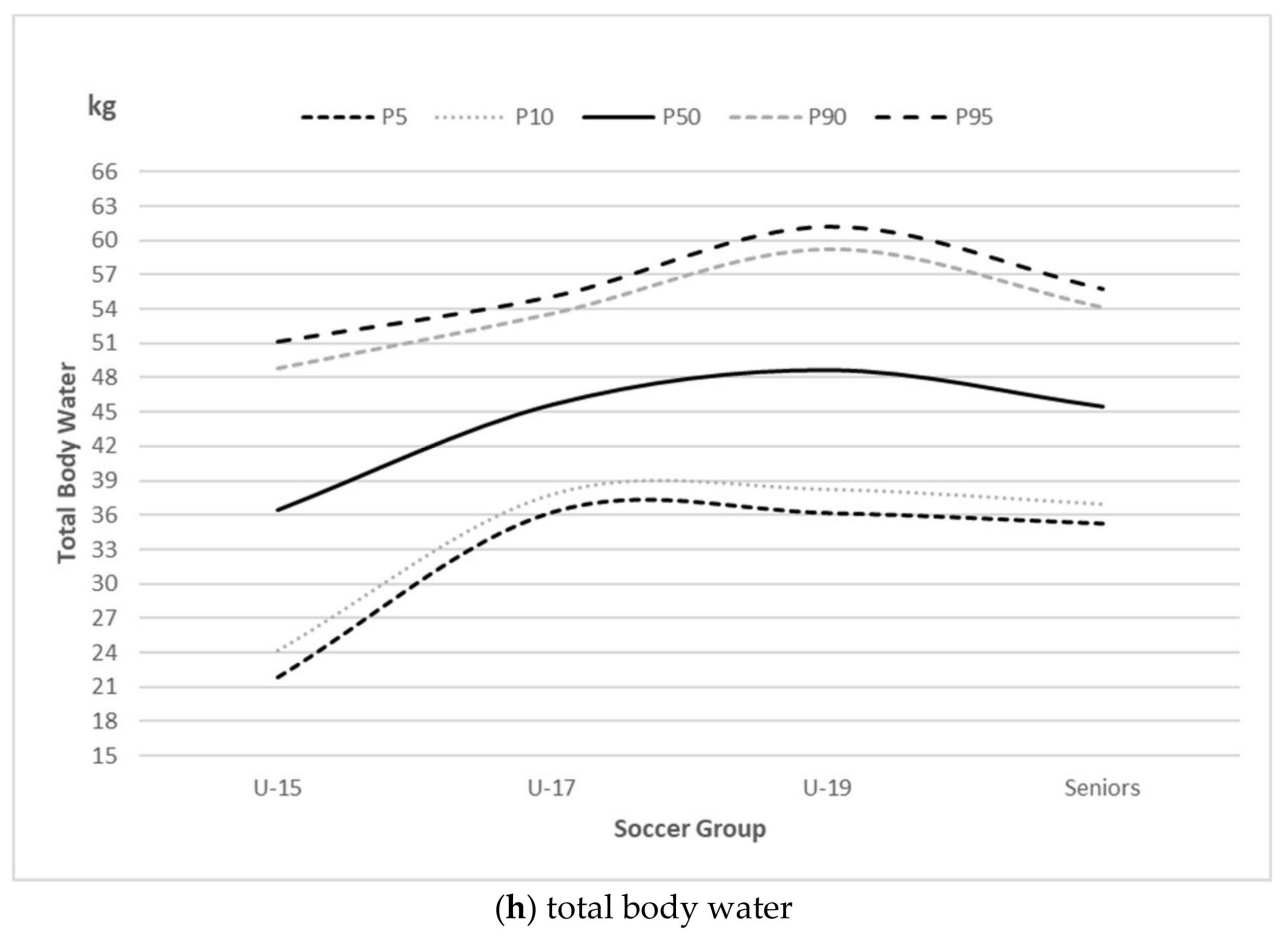
The 5th, 10th, 50th, 90th and 95th fitted centiles of (**a**) body height, (**b**) body weight, (**c**) body mass index, (**d**) basal metabolic rate, (**e**) fat free mass, (**f**) body fat mass, (**g**) skeletal muscle mass, (**h**) total body water for soccer groups.

The 5th, 10th, 50th, 90th and 95th fitted centiles of body height, weight, body mass index, skeletal muscle mass, percentage of body fat mass, fat-free mass, total body water and basal metabolic rate are presented in Table 4–d for U-15, U-17, U-19 and seniors, respectively. Figure 1a–h graphically also illustrates the 5th, 10th, 50th, 90th and 95th fitted centiles of anthropometric and body composition measures for the U-15, U-17, U-19 and seniors.

## 4. Discussion

This study aimed to investigate the morphological characteristics in four competitive age groups of soccer players, U-15, U-17, U-19, and seniors. Additionally, we aimed to show trends of body height, body weight, body mass index, skeletal muscle mass, percentage of body fat mass, fat-free mass, total body water and basal metabolic rate concerning the aforementioned competitive age groups. Ultimately, the present study aimed to display the optimal anthropometric and body composition profile of male soccer players across U-15, U-17, U-19, and seniors. 

The current study sample of soccer players tend to have larger body size compared to the previous review of body height and weight of Brazilian soccer players in U-17 from 1996 to 2006 (BHmean ranged from 173 to 177 cm; BWmean from 60 to 70 kg) [16]. The soccer players in our study in the U-15 and U-17 groups were also, on average, taller and heavier compared to the sample of Brazilian soccer players in Borges’s recent study (BHmean: 163.4 cm, 170.15 cm; BWmean: 50.46 kg, 62.73 kg, respectively) [17]. 

The age group a soccer player belongs to has an effect on his body composition, to generalize the outcomes of the present study. It has been revealed that the mean body height, body weight, body mass index, skeletal muscle mass, percentage of body fat mass, fat-free mass, total body water and basal metabolic rate of the U-15 soccer players was significantly lower than in the remaining groups of soccer players, U-17, U-19, and seniors. The larger body size for U-17 compared to U-15 soccer players was also confirmed by Da Silva et al.’s, and Matta et al.’s research [16,18]. Lower total body water and basal metabolic rate in the younger competitive age-related category were also previously found in [19]. Body composition dissimilarities were not found across U-17, U-19, and seniors, even though the mean body composition of the U-15 group was significantly different in from players in the remaining groups. Small body composition differences between the aforementioned competitive age-related soccer categories may have not been significant. Meanwhile, in the U-15 group, the impact of biological development is the greatest compared to the remaining competitive age ranks [18], which could have influenced inconsistent, non-linear changes in morphological characteristics across age-related soccer groups. In addition, the possible reason for failure to differentiate seniors’ body composition from the body composition of U-17 and U-19 could be the smaller sample size and wider age range of senior soccer players relative to the U-17 and U-19 groups. 

Although it has previously been reported that the percentage of body fat mass tends to decrease with age [9,10,20,21], our results suggest an increase in body size and composition with advancement in age-related soccer ranking. However, the relationship between age and the percentage of body fat may not be clear; the greater increase in fat-free mass may have changed the relative share of body fat mass in body mass, i.e., increasing the ratio of fat-free mass to body fat mass, because kilograms of body fat mass also tend to increase with age in our sample of soccer players. Milson et al. came to a similar conclusion [10]. Kilograms of fat-free mass increased for 29.1% from U-15 to U-17, but body fat mass did not reflect this increase.

The aforementioned inconsistency of gaining fat-free mass and body fat mass as the age of soccer players increases is better represented with cubic regression than linear in this sample of male soccer players. In spite of the inconsistency, age impacts fat-free mass and body fat mass in young males differently. Overall, the components of body composition, body height and weight are differently determined by the age groups of soccer players, where the magnitude of change in each morphological characteristic from U-15 to the senior category is not identical (Figure 1). The largest increment of body height, body weight, body mass index, skeletal muscle mass, fat-free mass, total body water, and basal metabolic rate has been shown to be from U-15 to U-17, due to the impact of biological development [22]. Therefore, none of the measured morphological characteristics have been reported to tend, on average, to linearly change with a higher ranking in male soccer players, in this study (~5% lower R^2^ for linear models). In addition, variation in body composition and body height and weight tend to change with age-related soccer rank in males.

The morphological characteristics were only explained with the age group of soccer players because we aimed to disclose age influence. For that reason, the determination of body composition and body height and weight according to age group of soccer players is not highly reliable (R^2^ ranged from 0.06 to 0.35). However, age in youth, or biological development, is not the only contributor to changes in body composition, but also lifestyle [21,23,24].

This is a preliminary study that, although it has a small sample, opens up an interesting path in the world of monitoring and control of footballers’ anthropometry. Studies with a much larger population are needed to ratify these first results. Moreover, a limitation of the current study is the fact that we did not use the proposed equations for body composition variables considering highly trained athletes or maturation effects [25,26,27]. Nevertheless, strength and conditioning programs to achieve specific body composition according to age profiles can be applied in the search for an advantage in physique in the respective age groups.

The current study increases the knowledge regarding the body composition characteristics of young and adult soccer players and how these characteristics change throughout the normal development and training process. In summary, the novelty of this study could be the fact that body fat mass remains stable and similar across all age groups as well as in senior squads. This fact shows that body composition is an important component of the athletes’ individualized and periodized training process across all age groups.

## 5. Conclusions

According to the results from this study, the required body composition profile of soccer players in older categories is different from the advisable profile in U-15, where younger soccer players tend to have lower absolute values of morphological characteristics. Reproduction of the morphological characteristics profile of male soccer players for four age-related soccer players’ categories in terms of charts and tables could give further insights into advisable and optimal morphological characteristics in U-15, U-17, U-19, and seniors, which could help in the selection process, due to its demonstrated relevance.

Models for forecasting the morphological characteristics in differently ranked soccer players require the involvement of more explanatory variables in order to achieve more reliable results, i.e., to estimate morphological characteristics’ true values within a diminished confidence interval and standard error of estimate. In addition, age also influences relationships between assessed morphological components in male soccer players, but this is beyond the scope of this paper.

## Figures and Tables

**Table 1:** Body height, weight and composition across age-related categories of soccer players.

Measures	U-15 (*n* = 152)	U-17 (*n* = 154)	U-19 (*n* = 61)	Seniors (*n* = 27)
Age (years)	13.7 ± 1.9	16.5 ± 0.5	18.2 ± 0.4	22.5 ± 4.9
BH (cm)	168.1 ± 14.1 *	180.6 ± 6.5	180.7 ± 6.6	179.7 ± 6.8
BW (kg)	56.5 ± 1278 *	70.5 ± 8.0	73.0 ± 8.4	72.4 ± 7.9
BMI (kg/m^2^)	19.7 ± 2.4 *	21.7 ± 2.0	22.3 ± 2.0	22.4 ± 1.8
MM (kg)	27.5 ± 7.5 *	36.0 ± 4.1	37.2 ± 4.5	36.6 ± 4.3
BF (kg)	7.1 ± 3.8	7.1 ± 3.0	7.7 ± 3.4	8.5 ± 3.0
BF (%)	12.9 ± 6.8 ^#^	10.0 ± 3.6	10.4 ± 3.9	11.6 ± 3.7
FFM (kg)	49.1 ± 12.7 *	63.4 ± 6.9	65.3 ± 7.5	64.0 ± 7.1
TBW (kg)	36.3 ± 9.1 *	46.4 ± 5.0	46.9 ± 7.6	46.9 ± 5.2
BMR (kcal/day)	1437.7 ± 269.5 *	1738.8 ± 148.3	1779.8 ± 163.3	1752.2 ± 153.1

Values are mean ± standard deviations; BH—body height; BW—body weight; BMI—body mass index; MM—skeletal muscle mass; BF—body fat mass; FFM—fat-free mass; TBW—total body water; BMR—basal metabolic rate; * U-15 significantly different from the remaining groups; ^#^ U-15 significantly different from U-17 and U-19.

**Table 2:** Correlation between study outcomes and age category.

Outcomes	Age Category
BH (cm)	0.49 **
BW (kg)	0.61 **
BMI (kg/m^2^)	0.48 **
MM (kg)	0.62 **
BF (kg)	0.10 *
BF (%)	−0.20 **
FFM (kg)	0.61 **
TBW (kg)	0.60 **
BMR (kcal/day)	0.61 **

Values are Spearman’s rank correlation coefficient. BH—body height; BW—body weight; BMI—body mass index; MM—skeletal muscle mass; BF—body fat mass; FFM—fat-free mass; TBW—total body water; BMR—basal metabolic rate; ** significant at *p* ≤ 0.01; * significant at *p* ≤ 0.05.

**Table 3:** Regression equations for the mean and SD of body height, weight, body mass index, skeletal muscle mass, percentage of body fat mass, fat-free mass, total body water and basal metabolic rate by competitive age category in male soccer players.

BIA Measure		Regression Equations	R^2^ (%)
BH (cm)	mean	131.10 + 51.66 × AC − 17.45 × AC^2^ + 1.88 × AC^3^	25.1
	SD	20.80 − 11.59 × AC + 1.98 × AC^2^	
BW (kg)	mean	35.45 + 25.42 × AC − 4.12 × AC^2^	33.5
	SD	15.89 − 7.27 × AC + 1.27 × AC^2^	
BMI (kg/m^2^)	mean	16.80 + 3.45 × AC − 0.52 × AC^2^	20.8
	SD	1.30 + 0.55 × 1/AC	
MM (kg)	mean	14.40 + 15.86 × AC − 2.63 × AC^2^	36.2
	SD	17.35 − 16.80 × AC + 6.39 × AC^2^ − 0.76 × AC^3^	
BF (%)	mean	17.92 − 6.24 × AC + 1.19 × AC^2^	6.3
	SD	9.01 − 4.77 × AC + 0.90 × AC^2^	
FFM (kg)	mean	27.24 + 26.57 × AC − 4.43 × AC^2^	35.7
	SD	29.29 − 28.44 × AC + 10.80 × AC^2^ − 1.29 × AC^3^	
TBW (kg)	mean	21.08 + 18.52 × AC − 3.10 × AC^2^	31.6
	SD	23.13 − 24.02 × AC + 9.55 × AC^2^ − 1.18 × AC^3^	
BMR (kcal/day)	mean	974.16 + 562.28 × AC − 93.72 × AC^2^	35.2
	SD	620.21 − 600.77 × AC + 228.66 × AC^2^ − 27.40 × AC^3^	

BH—body height; BW—body weight; BMI—body mass index; MM—skeletal muscle mass; BF—percentages of body fat mass; FFM—fat-free mass; TBW—total body water; BMR—basal metabolic rate; AC—age category (1—U-15; 2—U-17; 3—U-19; 4—seniors).

**Table 4:** The fitted centiles of body height (BH), body weight (BW), body mass index (BMI), skeletal muscle mass (MM), percentage of body fat mass (BF), fat-free mass (FFM), total body water (TBW) and basal metabolic rate (BMR) in (**a**) U-15, (**b**) U-17, (**c**) U-19 and (**d**) senior soccer players.

**(a) U-15**					
**Measure**	**5%**	**10%**	**50%**	**90%**	**95%**
BH (cm)	146.2	149.7	168.1	186.5	190.0
BW (kg)	37.4	40.5	56.8	73.0	76.2
BMI (kg/m^2^)	16.1	16.7	19.7	22.8	23.4
MM (kg)	15.5	17.5	168.1	37.8	39.7
BF (%)	2.9	4.5	12.9	21.2	22.8
FFM (kg)	28.9	32.2	49.4	66.6	69.9
TBW (kg)	21.9	24.2	36.5	48.8	51.1
BMR (kcal)	1010.2	1079.7	1442.7	1805.8	1875.3
**(b) U-17**					
**Measure**	**5%**	**10%**	**50%**	**90%**	**95%**
BH (cm)	167.0	169.2	180.6	192.0	194.2
BW (kg)	54.0	56.5	69.8	83.1	85.6
BMI (kg/m^2^)	15.7	16.7	21.6	26.6	27.5
MM (kg)	27.8	29.0	180.6	42.2	43.4
BF (%)	3.3	4.4	10.2	16.0	17.1
FFM (kg)	48.9	51.1	62.7	74.2	76.4
TBW (kg)	36.3	37.8	45.7	53.6	55.1
BMR (kcal)	1443.6	1488.6	1723.8	1959.1	2004.1
**(c) U-19**					
**Measure**	**5%**	**10%**	**50%**	**90%**	**95%**
BH (cm)	171.2	172.7	180.7	188.6	190.1
BW (kg)	61.0	63.2	74.6	86.0	88.2
BMI (kg/m^2^)	15.2	16.4	22.5	28.6	29.7
MM (kg)	29.0	30.5	180.7	46.1	47.6
BF (%)	4.4	5.3	9.9	14.5	15.4
FFM (kg)	49.8	52.6	67.1	81.5	84.3
TBW (kg)	36.2	38.2	48.7	59.2	61.2
BMR (kcal)	1483.3	1537.0	1817.5	2098.1	2151.8
**(d) Senior Soccer Players**					
**Measure**	**5%**	**10%**	**50%**	**90%**	**95%**
BH (cm)	164.7	167.1	179.7	192.3	194.7
BW (kg)	53.5	56.4	71.2	86.0	88.8
BMI (kg/m^2^)	13.7	15.1	22.3	29.5	30.9
MM (kg)	27.3	28.7	179.7	42.9	44.2
BF (%)	3.8	5.1	12.0	18.8	20.2
FFM (kg)	45.6	48.3	62.6	76.9	79.7
TBW (kg)	35.3	36.9	45.5	54.1	55.7
BMR (kcal)	1423.5	1471.7	1723.8	1975.8	2024.1

Values are means.

## Data Availability

Data generated and analyzed during this study are included in this article. Additional data are available from the corresponding author on request.
